# IFN-γ-Inducible Irga6 Mediates Host Resistance against *Chlamydia trachomatis* via Autophagy

**DOI:** 10.1371/journal.pone.0004588

**Published:** 2009-02-26

**Authors:** Munir A. Al-Zeer, Hesham M. Al-Younes, Peter R. Braun, Jens Zerrahn, Thomas F. Meyer

**Affiliations:** 1 Department of Molecular Biology, Max Planck Institute for Infection Biology, Berlin, Germany; 2 Department of Immunology, Max Planck Institute for Infection Biology, Berlin, Germany; 3 Department of Biological Sciences, Faculty of Sciences, University of Jordan, Amman, Jordan; Technical University Munich, Germany

## Abstract

Chlamydial infection of the host cell induces Gamma interferon (IFNγ), a central immunoprotector for humans and mice. The primary defense against *Chlamydia* infection in the mouse involves the IFNγ-inducible family of IRG proteins; however, the precise mechanisms mediating the pathogen's elimination are unknown. In this study, we identify Irga6 as an important resistance factor against *C. trachomatis*, but not *C. muridarum*, infection in IFNγ-stimulated mouse embryonic fibroblasts (MEFs). We show that Irga6, Irgd, Irgm2 and Irgm3 accumulate at bacterial inclusions in MEFs upon stimulation with IFNγ, whereas Irgb6 colocalized in the presence or absence of the cytokine. This accumulation triggers a rerouting of bacterial inclusions to autophagosomes that subsequently fuse to lysosomes for elimination. Autophagy-deficient Atg5−/− MEFs and lysosomal acidification impaired cells surrender to infection. Irgm2, Irgm3 and Irgd still localize to inclusions in IFNγ-induced Atg5−/− cells, but Irga6 localization is disrupted indicating its pivotal role in pathogen resistance. Irga6-deficient (Irga6−/−) MEFs, in which chlamydial growth is enhanced, do not respond to IFNγ even though Irgb6, Irgd, Irgm2 and Irgm3 still localize to inclusions. Taken together, we identify Irga6 as a necessary factor in conferring host resistance by remodelling a classically nonfusogenic intracellular pathogen to stimulate fusion with autophagosomes, thereby rerouting the intruder to the lysosomal compartment for destruction.

## Introduction

Chlamydiae are obligate intracellular pathogens that cause a variety of infections in humans and animals. *Chlamydia trachomatis* is a primarily human pathogen associated with common sexually transmitted diseases and trachoma [1]. Chlamydiae undergo a unique biphasic developmental cycle that alternates between extracellular infectious, metabolically inert elementary bodies (EBs) and the intracellular non-infectious, metabolically active, multiplying reticulate bodies (RBs) [2], [3]. Bacteria enter the host cell and survive within a membrane-bound vacuole, termed the inclusion, in which they ensure their successful propagation by avoiding fusion with lysosomes [4], [5]. Non-fusogenity with lysosomes is controlled by the mode of cellular uptake [6], [7] and chlamydial protein factors [8], [9].

Gamma interferon (IFNγ) plays a central role in innate immunity against intracellular pathogens. It induces the expression of more than 1,200 genes, a number of which include effectors that function to eradicate pathogens from host cells. Their activation leads to a depletion of the tryptophan (Trp) pool [10], production of toxic nitric oxide [11], deprivation of intracellular iron pools [12] and induction of host autophagy [13], [14]. Among the genes highly induced by IFNγ are the immunity-related GTPases (IRGs; also known as small p47 GTPases), reviewed in [1], [15]–[18].

Recent work has indicated the involvement of several mouse IRG proteins in the growth regulation of pathogens within IFNγ-induced host cells: for instance, Irgm1 stimulates IFNγ-induced control of *Mycobacterium tuberculosis* and *Trypanosoma cruzi* in macrophages [19] and Irgm3 regulates IFNγ-induced control of *C. trachomatis* in fibroblasts [20]. Irgm3-, Irgd- and Irgm1-knockout mice displayed reduced resistance to several bacterial and protozoan pathogens despite an immune response and IFNγ production [21], [22]. This strong correlation between loss of resistance in intact mice and loss of IFNγ-induced control in cultured host cells suggests eliminating pathogens from host cells is a major function of these proteins.

IRG proteins localize predominantly to the endoplasmic reticulum (ER) (Irga6 and Irgm3), the Golgi (Irga6, Irgm1 and Irgm2), the plasma membrane and in nascent pathogen-containing vacuoles or phagosomes [reviewed in 16–18]. The localization of IRGs to pathogen-containing vacuoles in host cells suggests that they restrict pathogen growth by vacuole processing. IRGs have been shown to drive vacuole acidification and fusion with lysosomes in *M. tuberculosis* [19], to disrupt the vacuolar membrane in *Toxoplasma gondii* [23], [24] and to eliminate mycobacteria containing vacuoles through regulating IFNγ-induced autophagy [13], [14].

Autophagy is an evolutionary conserved lysosomal degradation pathway that maintains cellular homeostasis and selectively disposes of intracellular pathogens [13], [25], [26]. Not only intracellular pathogens residing in the cytosol, but also pathogens residing in membranous or intravacuolar compartments are sequestered into autophagosomes for degradation in autolysosomes, as shown for *T. gondii* [23], [27].

Chlamydiae exhibit a wide range of host tropism that has been linked to differences in immune responses elicited by IFNγ [28]. In human cells, IFNγ can effectively suppress growth of *C. trachomatis* and *C. muridarum* by activating indolamine dioxygenase, which deprives both of essential Trp. However, in murine genital epithelial cells (MECs), IFNγ can restrict growth of *C. trachomatis*, but not *C. muridarum* [28]. Growth inhibition of *C. trachomatis* in mouse cells is Trp depletion independent and is largely attributed to the IFNγ-inducible IRGs. Little is known about the cellular functions of mouse IRGs in host resistance against *Chlamydia* species. A role for Irga6 in controlling pathogen growth upon IFNγ stimulation in MECs was demonstrated by RNA silencing of Irga6, which led to increased *C. trachomatis* survival [28]. However, the major effector mechanism(s) by which IRGs control chlamydial infection remain elusive.

Here we investigated the immunoprotective role of IRGs in *C. trachomatis* infection of murine cells and in the IFNγ-insensitive mouse strain *C. muridarum*. We show that *C. trachomatis* growth is arrested by the development of early inclusions with autolysosomal features. In contrast, *C. muridarum* inclusions remained segregated from lysosomes and autophagosomes. Subcellular analysis revealed that *C. trachomatis* inclusions sequestered Irga6, Irgd, Irgm2 and Irgm3 in response to IFNγ blocking chlamydial growth. However, autophagy-deficient cells tolerated *C. trachomatis* infection despite an accumulation of Irgd, Irgm2 and Irgm3 at inclusions. Strikingly, Irga6 did not associate with inclusions in autophagy-deficient cells. In addition, Irga6−/− MEFs did not respond to IFNγ and did not restrict *C. trachomatis* growth, although Irgd, Irgm2 and Irgm3 localized to inclusions. Thus, our data indicate that Irga6 modifies the inclusion membrane to mediate fusion with autophagosomes as a mechanism to dispose of *C. trachomatis*.

## Results

### IFNγ negatively affects the growth of *C. trachomatis*, but not of *C. muridarum* in MEFs

**Figure 1:**
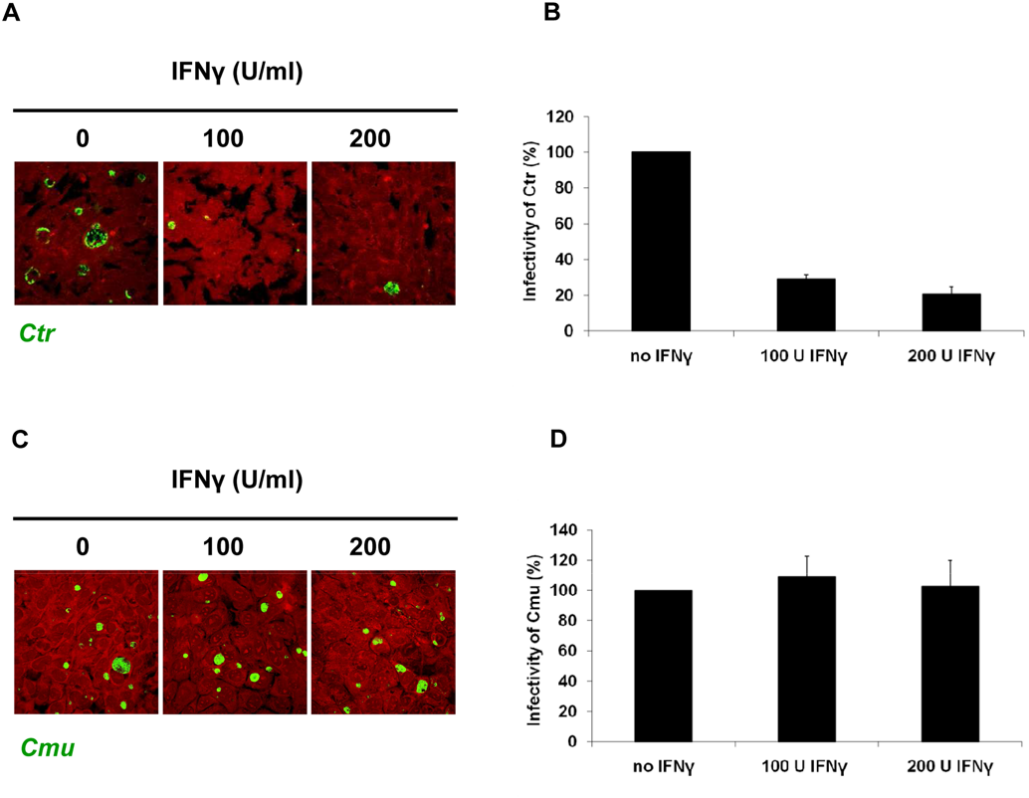
IFNγ-induced inhibition of *C. trachomatis* growth in WT MEFs. Host cells were infected for 48 h with *C. trachomatis* or *C. muridarum* (MOI 1) and simultaneously treated with 100 U or 200 U/ml IFNγ or left untreated (control). (A) and (C) Immunofluorescence micrographs of MEFs infected with *C. trachomatis* or *C. muridarum*, respectively, stained with *Chlamydia*-IMAGEN kit. Chlamydiae (green), Host cells (red). Cytokine treatment resulted in a low number of detectable small inclusions in *C. trachomatis* infected cells only. Images taken using the same magnification. (B) and (D) Influence of IFNγ on development of infectious progeny. The yield of *C. trachomatis* (B), but not *C. muridarum* (D), infectious progeny decreased considerably upon IFNγ stimulation, Infectivity percentage calculated as follows: IFU/ml estimated for each treated monolayer / IFU/ml of control cells ×100. Infectivity expressed as a percentage of control cells ±standard deviation (SD) from three independent experiments (n = 3). WT, wild type; *Ctr*, *C. trachomatis*; *Cmur*, *C. muridarum*.

IFNγ is a critical mediator for controlling chlamydial infection. To assess the effect of IFNγ on chlamydial growth in mouse embryonic fibroblasts (MEFs), cells were infected with *C. trachomatis* LGV L2 or *C. muridarum* at a multiplicity of infection (MOI) of 1 for 2 h. Following 48 h incubation in the presence of IFNγ, cells were immunostained and analyzed by microscopy. IFNγ treatment resulted in much smaller and substantially reduced numbers of *C. trachomatis* inclusions (Figure 1A). Similar results were obtained from the pretreated cells with IFNγ. Furthermore, a reduction of infectious *C. trachomatis* EBs of more than 70% as compared to non-treated infected MEFs (Figure 1B) was found. In contrast, IFNγ affected neither inclusion development nor formation of infectious progeny of *C. muridarum* (Figure 1C and D). Thus, distinct chlamydial species exhibit differential sensitivities to IFNγ in host resistance: Growth of *C. trachomatis* in MEFs is arrested in response to IFNγ, whereas growth of *C. muridarum* is not affected in response to IFNγ.

### Irga6, Irgd, Irgm2 and Irgm3 interact specifically with early *C. trachomatis* inclusions in response to IFNγ

**Figure 2:**
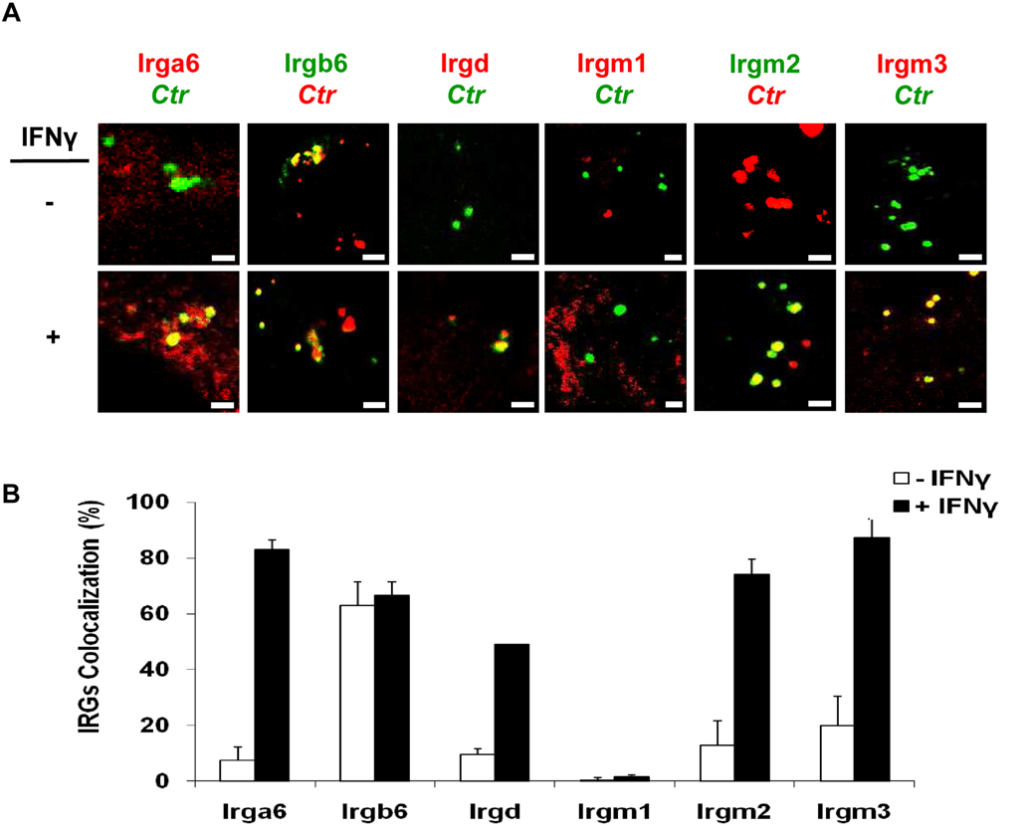
IFNγ triggers accumulation of Irga6, Irgd, Irgm2 and Irgm3 at early inclusions of *C. trachomatis*. (A) Double immunofluorescence labelling of IRGs and *C. trachomatis* in MEFs stimulated for 24 h with 100 U/ml IFNγ and then infected for 3 h with *C. trachomatis* (MOI 5). IFNγ untreated MEFs were similarly infected. Upon IFNγ induction Irga6, Irgd, Irgm2 and Irgm3 localized to inclusions. Irgb6 colocalized strongly with inclusions in treated as well as untreated cells, whereas Irgm1 localization was minimal. (B) For quantification, around 300 bacterial inclusions were examined for each IRG in cytokine-treated or untreated MEFs. Colocalization expressed as a mean percentage: for each treatment, number of IRG +ve inclusions / total number of inclusions ×100. Error bars ±SD, n = 3. Scale bar represents 4 µm.

Since IFNγ can upregulate IRG expression in *C. trachomatis* infected murine cells [28], we investigated the involvement of IRGs in IFNγ-mediated inhibition of chlamydial growth. Untreated and IFNγ-pretreated MEFs were infected with *C. trachomatis* or *C. muridarum* (MOI 5) in the presence of IFNγ. Three hours post infection (h p.i.) cells were processed for indirect immunofluorescence to analyze colocalization of early chlamydial inclusions with IRGs (Figure 2A). Upon IFNγ treatment *C. trachomatis* inclusions colocalized to a high degree with IRGs: Irga6 (83%), Irgd (49%), Irgm2 (74%) and Irgm3 (87%). Surprisingly, Irgb6 also colocalized with inclusions in both treated (63%) and untreated cells, whereas Irgm1 colocalization was minimal (Figure 2B). However, no localization of IRGs to *C. muridarum* inclusions was detected (Figure S1A). Thus, IFNγ stimulation leads to a specific modification of *C. trachomatis* inclusions by recruiting four different IRGs.

### IFNγ stimulates *C. trachomatis* elimination by lysosomal fusion with early inclusions

**Figure 3:**
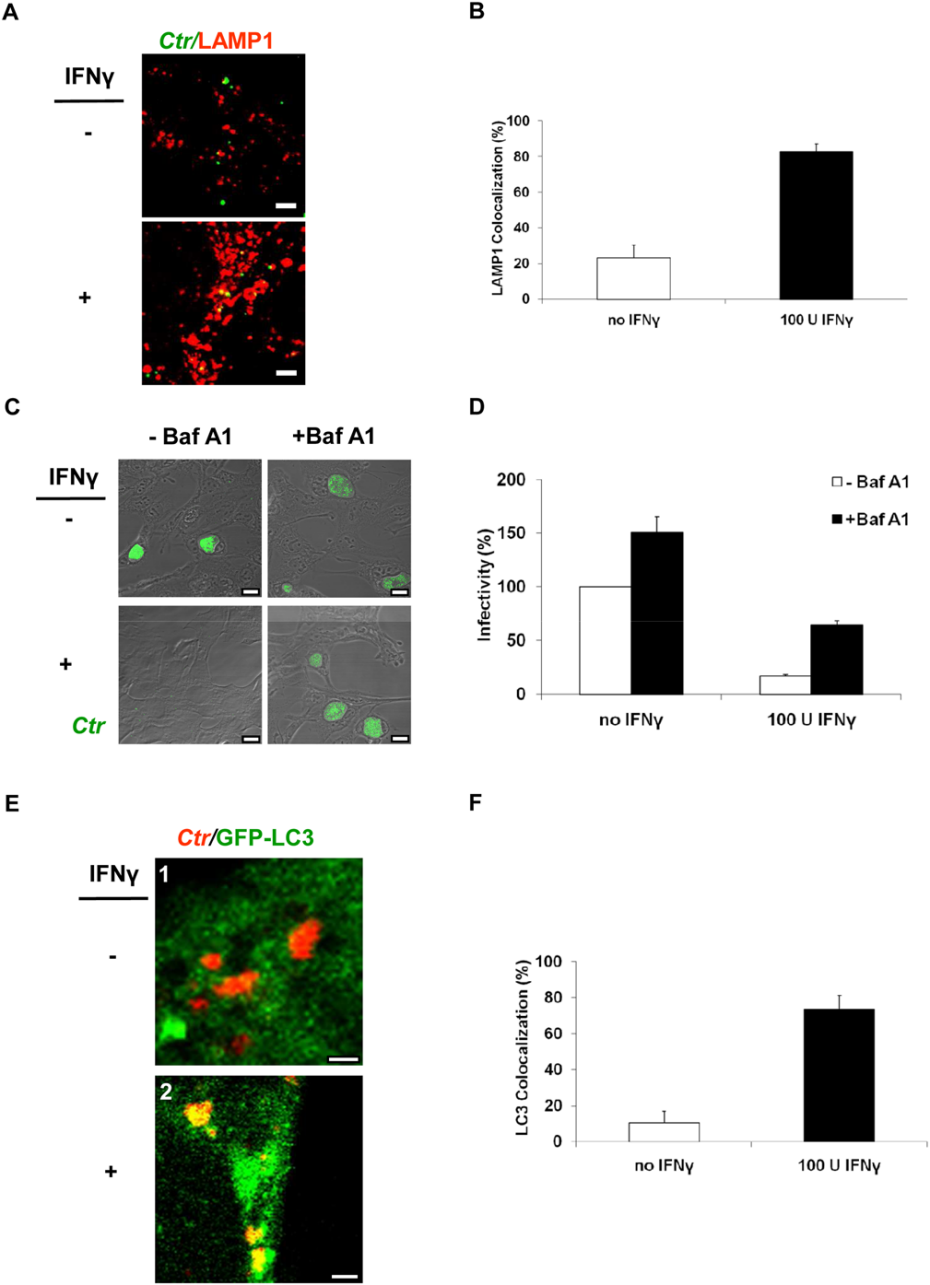
IFNγ stimulation induces interaction of lysosomes and autophagosomes with early *C. trachomatis* inclusions in MEFs. (A) and (B) MEF monolayers were prestimulated for 24 h with 100 U/ml IFNγ and then infected with *C. trachomatis* as described in Figure 2. IFNγ untreated control MEFs were similarly infected. (A) Double immunofluorescence labelling 3 h p.i. revealed that IFNγ stimulated the association of the lysosomal marker LAMP1 (red) with *C. trachomatis* (green) inclusions (compare panel 2 with 1). (B) Percentages of colocalization with LAMP1. (C) Inhibition of lysosomal acidification by 100 nM Baf A1 stopped the IFNγ-mediated inhibition of *C. trachomatis* inclusion growth. (D) Baf A1 attenuated the IFNγ-induced reduction of *C. trachomatis* infectivity in MEFs. Infectivity assays were performed as in Figure 1B. Baf A1 led to an increase in the yield of infectious progeny in IFNγ-treated MEFs 48 h p.i. (E) and (F) IFNγ induces localization of autophagosomes to inclusions. MEFs were transfected for 24 h with the autophagosome membrane marker GFP-LC3 and then exposed to 100 U/ml IFNγ for an additional 24 h. Next, cells were infected with *C. trachomatis* (MOI 5) for 8 h. IFNγ-untreated control cells were similarly infected 48 h post-transfection. LC3 (green) localized to bacterial inclusions (red) in response to IFNγ stimulation (compare panel 2 with 1 in E). (F) Quantification of GFP-LC3-positive *C. trachomatis* inclusions in the presence or absence of IFNγ. Around 300 bacterial inclusions were examined for LAMP1 or LC3 sequestration. Colocalization expressed as a mean percentage: for each treatment, number of LAMP1 or LC3 +ve inclusions, respectively / total number of inclusions ×100.Scale bars in (A), (C) and (E) represent 2, 20 and 1 µm, respectively. Error bars ±SD, n = 3.

We next investigated whether IRG-positive *C. trachomatis* inclusions are directed to lysosomes for degradation. First we monitored colocalization of inclusions with the lysosomal marker LAMP1 at 3 h p.i. In control, untreated MEFs, only 20% of inclusions colocalized with LAMP1. In contrast, LAMP1 colocalization in inclusions of IFNγ-treated cells was increased 4-fold (Figure 3A and B). Consistent with an ability to grow in the presence of IFNγ, no LAMP1 colocalization with *C. muridarum* inclusions was observed in untreated or IFNγ-treated cells (Figure S2A and B). To confirm that IFNγ-induced phagosome-lysosome fusion eliminates *C. trachomatis*, we studied the effect of inhibiting lysosomal acidification with bafilomycin A1 (Baf A1), a specific vacuolar H^+^-ATPase inhibitor. Growth of *C. trachomatis* inclusions was rescued and formation of infectious EBs increased upon Baf treatment (Figure 3C and D). These data clearly indicate that lysosomal fusion elicited by IFNγ eliminates chlamydial inclusions.

### IFNγ-induced autophagy is required for the elimination of *C. trachomatis*

IFNγ can induce autophagy to eliminate intracellular pathogens [13], [14]. We hypothesized that IFNγ induces the interaction of *C. trachomatis* inclusions with autophagosomes to reroute the intruder to the lysosomal compartment for destruction; therefore, we transfected MEFs with the autophagosome-associated marker GFP-LC3, a GFP fusion with the microtubule-associated protein light chain 3 (LC3). Then, MEFs were either infected with *C. trachomatis* or *C. muridarum* in the presence or absence of IFNγ. In contrast to IFNγ-untreated cells, GFP-LC3 colocalized strongly to *C. trachomatis* inclusions (74%) in IFNγ treated cells (Figure 3E and F). However, minimal GFP-LC3 colocalization was observed in *C. muridarum* inclusions (Figure S2C and D). Taken together with our LAMP1 data, these findings clearly suggest the nature of *C. trachomati*s inclusions upon IFNγ stimulation is autolysosomal.

**Figure 4:**
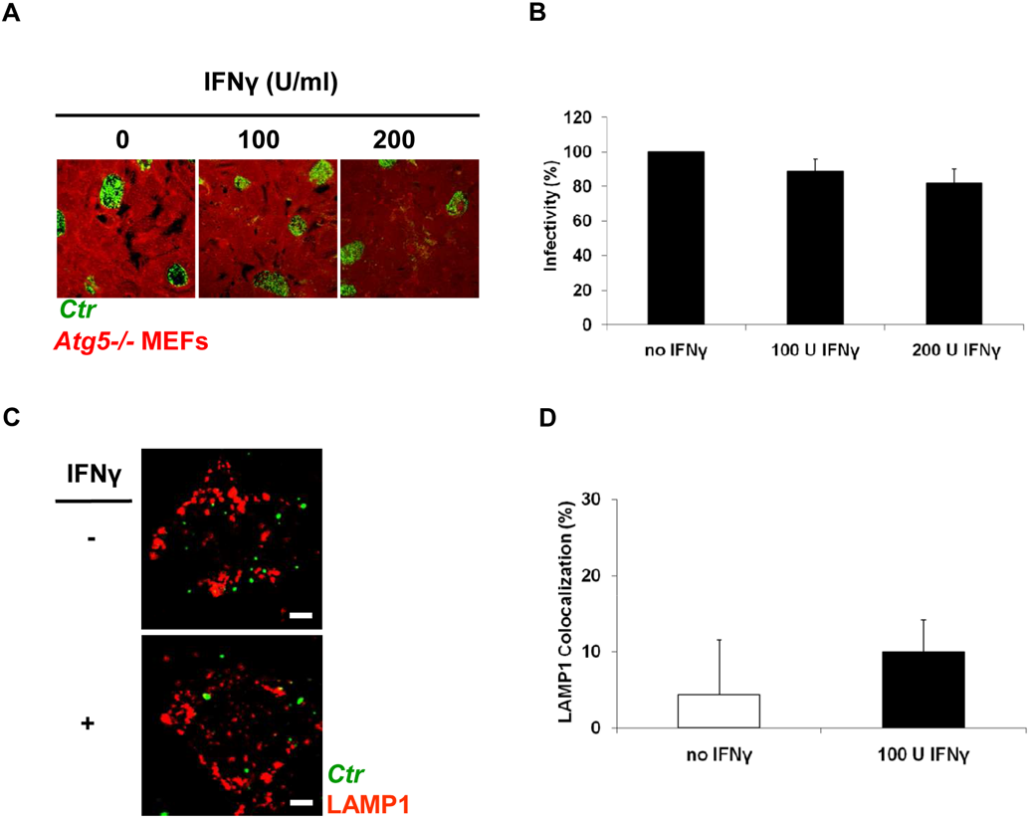
IFNγ cannot induce lysosomal fusion with early *C. trachomatis* inclusions to suppress bacterial growth in autophagy-lacking (Atg5−/−) MEFs. (A) Normal development of inclusions (green) was observed in Atg5−/− MEFs (red), despite exposure to either 100 U or 200 U IFNγ/ml. Knockout cells were IFNγ treated, infected, and stained as in Figure 1. (B) Infectivity titration assays onto fresh HeLa cells revealed similar amounts of infectious progeny in IFNγ-treated and IFNγ-untreated Atg5−/− MEFs. Infectivity expressed as a percentage normalized to control (C) *C. trachomatis* inclusions avoid interaction with lysosomes in autophagy-defective Atg5−/− MEFs despite IFNγ induction. IFNγ treated and untreated Atg5−/− MEFs were infected as in Figure 3A and B. Double immunolabeling demonstrated no recruitment of lysosomes to inclusions [bacteria (green) and LAMP1 (red)] (D) Quantification of LAMP1-positive chlamydial inclusions revealed insignificant colocalization rates. Around 300 bacterial inclusions were examined. Colocalization expressed as a mean percentage: for each treatment, number of LAMP1 inclusions / total number of inclusions ×100. Images in (A) were taken under the same magnification, while scale bars in (C) represent 3 µm. Error bars ±SD, n = 3.

To further support our hypothesis, we used Atg5-deficient (Atg5−/−) MEFs (Atg, autophagy-related). Atg5 is a crucial factor in autophagosome formation as its deletion prevents their appearance [29]. We infected Atg5−/− MEFs with either *C. trachomatis* or *C. muridarum* (MOI 1) for 48 h in the presence or absence of IFNγ. IFNγ treatment did not affect chlamydial growth as inclusion size and recovery of infectious progeny in these cells were comparable to those of untreated knockout cells (Figure 4A and B) and WT MEFs. The completion of chlamydial development in cells devoid of autophagy shows that host cell resistance to chlamydial inclusions depends on their fusion with autophagosomes. Furthermore, subcellular analysis of cultures at 3 h p.i. with LAMP1 revealed that lysosomal fusion is blocked in Atg5−/− MEFs (Figure 4C and D). Thus, fusion of *C. trachomatis* inclusions with autophagosomes is a prerequisite for lysosomal degradation.

### Association of Irga6 with early chlamydial inclusions is blocked in Atg5−/− MEFs in response to IFNγ

**Figure 5:**
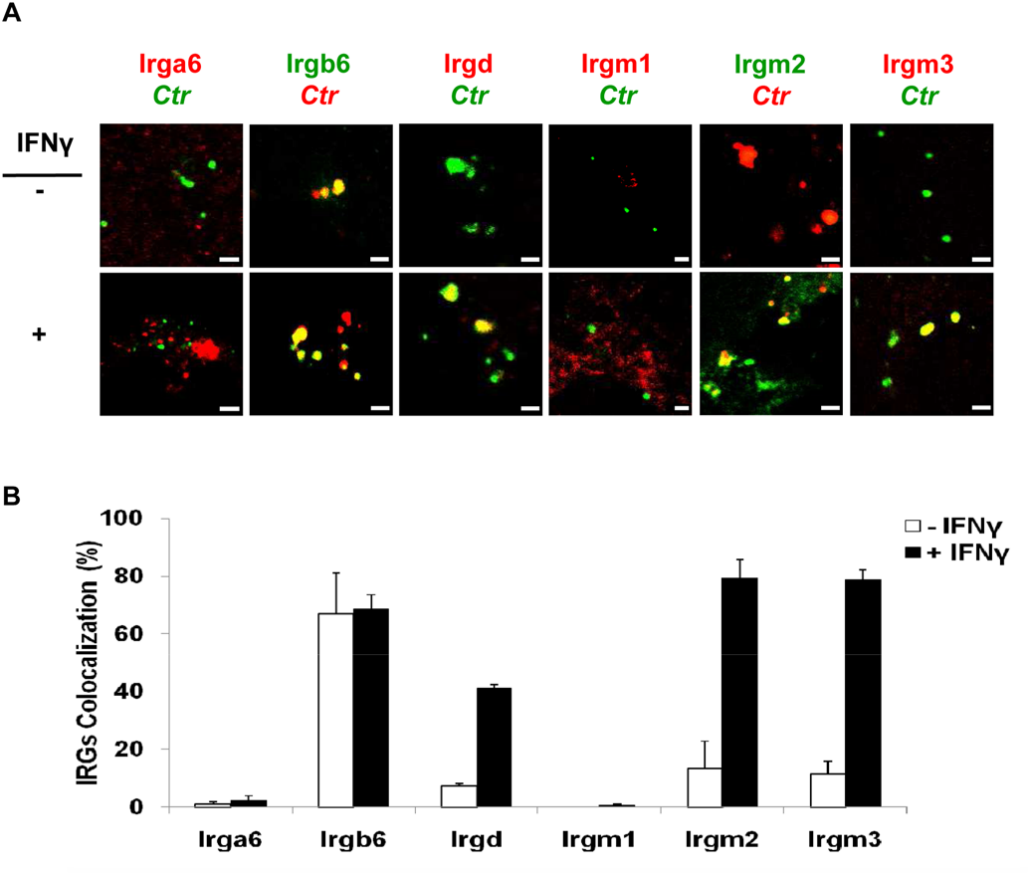
Absence of Irga6 colocalization at *C. trachomatis* early inclusions in IFNγ stimulated Atg5−/− MEFs. (A) and (B) IFNγ treated and untreated cells were infected with *C. trachomatis* as in Figure 2. (A) Confocal microscopic analysis of infected stimulated Atg5-knockout cells shows similar staining patterns for Irgb6, Irgd, Irgm1, Irgm2 and Irgm3 as in stimulated infected WT MEFs (Figure 2). Irga6 does not localize to the inclusion in IFNγ-stimulated and unstimulated Atg5-knockout cells. (B) Quantification of colocalization rates of IRGs with *C. trachomatis* inclusions. Around 300 bacterial inclusions were examined. Colocalization expressed as a mean percentage: for each treatment, number of IRG +ve inclusions / total number of inclusions ×100. Error bars ±SD, n = 3. Scale bar represents 4 µm.

To address the role of IFNγ-induced IRGs in the regulation of *C. trachomatis* survival, we examined the intracellular distribution of IRGs at 3 h p.i. in Atg5−/− MEFs. Surprisingly, upon IFNγ stimulation most bacterial inclusions colocalized with Irgd (41%), Irgm2 (80%) and Irgm3 (79%) (Figure 5A and B); results comparable to data obtained from IFNγ-stimulated WT MEFs (Figure 2). Strikingly, only 2% of inclusions colocalized with Irga6 (Figure 5B), as compared to 83% in IFNγ-treated WT MEFs (Figure 2B). As expected, IRG proteins did not colocalize with early inclusions of *C. muridarum* (Figure S1B). These results strongly suggest an important role for Irga6 in autophagy-mediated control of *C. trachomatis* infection.

### Absence of Irga6 stimulates growth of *C. trachomatis* and induces resistance against IFNγ-induced killing

**Figure 6:**
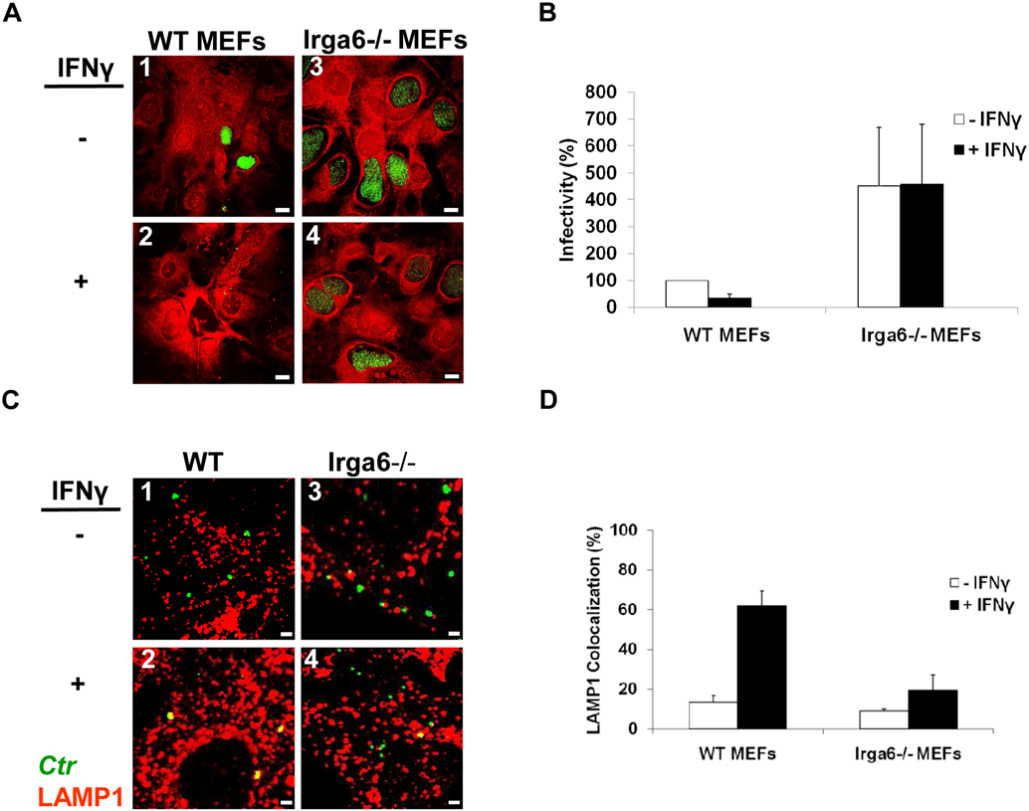
Absence of Irga6 enhances *C. trachomatis* replication and resistance against IFNγ-induced growth inhibition. (A) Deletion of Irga6 promotes intracellular growth of the bacterial inclusion. Unstimulated WT and Irga6-knockout MEFs were infected for 48 h (MOI 1). Micrographs show an increased inclusion size in Irga6-knockout cells (panel 3) compared to WT MEFs (panel 1). IFNγ (100 U/ml) stimulation did not suppress chlamydial growth in Irga6-deficient MEFs (panel 4) as in IFNγ-induced WT MEFs (panel 2). (B) Infectivity of bacteria in Irga6-knockout MEFs is 4-fold higher than in WT MEFs and is unaffected by IFNγ induction. Results depicted as mean percentage normalized to control. (C) Lack of appreciable lysosomal fusion with *C. trachomatis* inclusions in IFNγ-stimulated Irga6−/− MEFs (panel 4) unlike that in stimulated WT MEFs (panel 2). Cells were stimulated, infected and stained for bacteria (green) and LAMP1 (red) as in Figure 3A. Untreated WT and Irga6-knockout monolayers (panels 1 and 3, respectively) served as controls. (D) IFNγ did not considerably increase rates of LAMP1 localization to *C. trachomatis* inclusions in Irga6−/− MEFs, compared with that in WT cells. Around 300 bacterial inclusions were examined Colocalization expressed as a mean percentage: for each treatment, number of LAMP1 +ve inclusions / total number of inclusions ×100. Scale bar in (A) and (C) represent 40 and 4 µm, respectively. Error bars ±SD, n = 3.

To further analyze the role of Irga6 in IFNγ-mediated growth restriction of *C. trachomatis*, we examined Irga6-knockout (Irga6−/−) MEFs. Untreated Irga6−/− cells infected for 48 h with *C. trachomatis* generated larger inclusions and yielded a 4 to 5-fold increase in the number of infectious progeny, compared to untreated WT cells (Figure 6A and B). IFNγ treatment did not inhibit the growth of inclusions in Irga6−/− cells, but they were bigger than in control untreated WT MEFs (Figure 6A). Surprisingly, infected Irga6−/− MEFs were sensitive to IFNγ exposure, which resulted in partial destruction and loss of infected host cells of the monolayer. Therefore, the chlamydial infectivity measured in each sample had to be normalized to the surviving host cells, determined via an LDH release assay (data not shown). This data clearly indicated that IFNγ treatment of Irga6−/− cells did not reduce infectivity (Figure 6B).

Similar to our data from WT and Atg5−/− MEFs (Figure 2 and 5, respectively), IFNγ treatment of Irga6−/− MEFs at 3 h p.i. increased the association of Irgd, Irgm2 and Irgm3 with early bacterial inclusions (Figure S3A and S3B). As expected, no signal for Irga6 was detected in IFNγ-exposed or unexposed Irga6-deficient MEFs (Figure S3A). In contrast, no IRG proteins localized to *C. muridarum* inclusions (Figure S1C).

Next, we investigated whether enhanced propagation of *C. trachomatis* in Irga6−/− MEFs was connected with a lack of autolysosomal features of inclusions. Indeed, IFNγ treatment of Irga6−/− MEFs did not induce an association of LAMP1 with chlamydial inclusions (Figure 6C and D). Numbers of bacteria colocalizing with LAMP1 in IFNγ induced Irga6−/− cells were comparable to untreated WT MEFs (Figure 6C and D). Consistently, only approximately 1% of bacterial inclusions colocalized with GFP-LC3 in Irga6−/− cells. These results indicate that IFNγ cannot prevent *C. trachomatis* development in cells lacking Irga6. Thus, our data suggest a critical role for Irga6 in provoking anti-chlamydial effects. In contrast, despite their noticeable association with bacterial inclusions, Irgd, Irgm2 and Irgm3 do not play a critical role in anti-chlamydial defense. The loss of Irga6 renders cells incapable of capturing *C. trachomatis* in an autolysosome, thus enabling pathogen survival.

### Irga6 is important in the induction of autophagy independent of *C. trachomatis* infection

**Figure 7:**
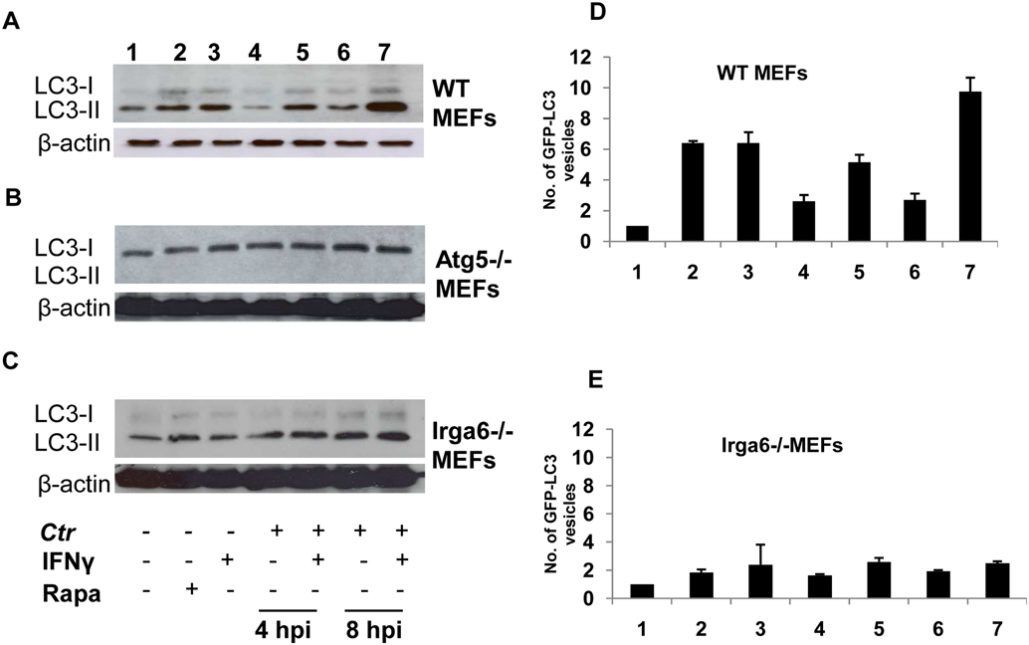
Irga6 plays a role in the regulation of the host autophagic machinery. (A–C) Anti-LC3 immunoblot analysis of total lysates from uninfected WT, Atg5−/− and Irga6−/− MEFs or from cultures infected for indicated time points. Some uninfected cell cultures were exposed to 100 nM Rapa for 3 h or to 100 U/ml IFNγ for 32 h. Other monolayers were pretreated with IFNγ for 24 h prior to infection and then infected in the presence of chemicals. 1–7 indicate the different treatments. Host β-actin was used to control equal loading of proteins. (A) Autophagy is induced by Rapa and IFNγ in WT cells, while infection alone does not stimulate autophagy, as indicated by the amount of LC3-II. (B) Defective autophagy in Atg5−/− MEFs was observed, indicated by absence of LC3 processing (C) Abolishment of Irga6 apparently impaired autophagy induction. Only very low amounts of cellular LC3 (LC3-II) can be detected in Irga6-knockout cells, compared to those in WT MEFs. (D)–(E) Quantification of GFP-LC3-vacoules under different conditions in WT (D) and Irga6−/− MEFs (E) treated as indicated for (A)–(C), 24 h after transfection. Around 100 cells were examined by immunofluorescence microscopy. Data normalized against control. Data show average numbers per cell within a set cytoplasmic area. Error bars ±SD, n = 3.

Is IFNγ capable of activating autophagy in MEFs and is Irga6 required for IFNγ-induced autophagy? To answer these questions, we used immunoblots to monitor the formation of early autophagosomal precursors and newly formed autophagosomes by following changes in LC3 expression. LC3 exists in two forms: the cytosolic LC3-I form, which has a molecular weight of approximately 18 kDa and the membrane-bound LC3-II form, with a molecular weight of 16 kDa [30]. LC3-II is bound to the membrane of nascent autophagosomes and correlates with the amount of LC3-positive autophagosomes [30]. In WT cells, LC3-II levels increased in response to IFNγ stimulation (Figure 7A, lane 3). Notably, levels of IFNγ-induced LC3-II were comparable to those induced by rapamycin (Rapa), a conventional inducer of autophagy (Figure 7A, lane 2). Infection with *C. trachomatis* for 4 h or 8 h did not influence LC3-II expression (Figure 7A, lanes 4 and 6, respectively). However, IFNγ treatment of infected WT MEFs induced an upregulation of LC3-II (Figure 7A, lanes 5 and 7). In general, levels of LC3 protein were substantially lower in Irga6−/− cells (Figure 7C) as compared to WT MEFs (Figure 7A). Moreover, IFNγ or Rapa treatment had a minimal stimulatory effect on LC3 expression and/or processing in Irga6−/− cells (Figure 7C, lanes 2, 3, 5 and 7). *C. muridarum* infection effectively suppressed an increase in LC3 levels upon IFNγ treatment (Figure S4A). Similarly, no increase in LC3 levels upon IFNγ treatment in *C. muridarum* infected Irga6−/− cells (Figure S4C) was observed. Atg5−/− cells did not process LC3 (Figure 7B and S4B), as expected for cells that are unable to form autophagosomes [31].

To confirm the importance of Irga6 in autophagy, we quantified the number of autophagosomes in the cytoplasm. In contrast to control untreated WT cells (Figure 7D), numbers of GFP-LC3-decorated structures per cell in infected and uninfected WT cells exposed to either IFNγ or Rapa were increased. In agreement with our immunoblot analysis, IFNγ or Rapa induction did not increase the amount of autophagosomes and we observed only low numbers of decorated vesicles in Irga6−/− cells (Figure 7E). These data further support a pivotal role for autophagy in *C. trachomatis* elimination and indicate a role for Irga6 as regulator of IFNγ-induced autophagy.

In summary, our data clearly demonstrates the ability of IFNγ to induce autophagy in nonphagocytic MEFs. More importantly, low LC3 expression and small numbers of autophagosomes in Irga6−/− cells indicate a role for Irga6 in the regulation of the IFNγ-induced autophagic pathway. Our results reveal Irga6 as an important factor in assigning chlamydial inclusions for degradation via autophagy.

## Discussion

Here we elucidate the mechanisms underlying IFNγ/IRGs-induced immunity to *C. trachomatis* in murine cells, demonstrating a pivotal role for Irga6 in mediating host resistance to infection via the induction of autophagy. We show that *C. trachomatis*, but not *C. muridarum*, inclusions strongly colocalized with 4 IRGs (Irga6, Irgd, Irgm2 and Irgm3) in response to IFNγ, whereas Irgb6 colocalized in the presence or absence of the cytokine. IFNγ also induced accumulation of the autophagosomal membrane marker LC3, and the lysosomal component LAMP1, suggesting a rerouting of *C. trachomatis* phagosomes to autolysosomes. However, autophagy-deficient MEFs (Atg5−/−) and WT cells with an impairment in lysosomal acidification surrendered to infection. While Irgb6, Irgd, Irgm2 and Irgm3 still localized to chlamydial inclusions in IFNγ-induced Atg5−/− cells, Irga6 localization was disrupted, indicating a pivotal role for Irga6 in resistance to the microbe. Strikingly, Irga6-deficient MEFs (Irga6−/−), in which chlamydial growth is enhanced compared to WT MEFs, showed no response to IFNγ even though all the major IRG proteins studied still localized to inclusions. Thus, Irga6 constitutes a critical resistance factor against *C. trachomatis* infection in IFNγ-induced MEFs that remodels a classically non-fusogenic intracellular pathogen vacuole, stimulating fusion with autophagosomes and directing the intruder to the lysosomal compartment for destruction.

Autophagy reroutes cytoplasmic material and organelles internalized into autophagosomes to lysosomes, culminating in the formation of autolysosomes and degradation of their cargo [30], [32] [33]. Similarly, this process can induce pathogen eradication. Here we showed IFNγ stimulates localization of LC3 and the lysosomal marker LAMP1 to *C. trachomatis*, but not *C. muridarum*, inclusions in WT MEFs. Despite the presence of IFNγ, autophagosome-deficient MEFs were highly permissive to *C. trachomatis* replication in a compartment disconnected from lysosomes, confirming an involvement of autophagy that leads to lysosomal degradation of the bacteria. Similarly, a study in macrophages showed that IFNγ stimulates recruitment of LC3 and LAMP1 to the *M. tuberculosis* compartment and induces autophagy to inhibit bacterial viability [13]. In addition, the induction of structures carrying LC3 close to disrupting *T. gondii* vacuoles in IFN-γ-induced astrocytes has been reported [24]. IFNγ therefore represents a novel means to counteract the non-fusogenicity of the *C. trachomatis* inclusion by remodelling it into a compartment with autophagic characteristics to prompt fusion with lysosomes for degradation.

In contrast to our results, a previous study implied that IFNγ-mediated suppression of *C. trachomatis* in MECs was not the result of fusion with lysosomes, but due to a reduction of lipid trafficking to inclusions [28]. This discrepancy can be explained by the fact that our LAMP1 analysis was done at 3 h p.i., a time at which most *C. trachomatis* early inclusions strongly colocalized with LAMP1. In contrast, Nelson and coworkers examined LAMP1 colocalization to 24 h-old inclusions, which probably represent the 20% of *C. trachomatis* inclusions that might partially have survived the IFNγ-mediated immunity. Nevertheless, we cannot dismiss a role for lipid and nutrient, or indeed other vacuolar trafficking in the IFNγ-mediated suppression of Chlamydial growth. For instance, it has been shown that Irga6 interacts with the microtubule-binding protein hook3; therefore, it is tempting to consider that Irga6 participates in the modulation of intracellular membrane-dependent processes [34], [46] like vesicular trafficking and interactions with the pathogen-containing vacuolar membrane components.

Several studies have implicated the IRG family of proteins in growth regulation of intracellular pathogens [13], [14], [19], [35]. For instance, Irga6 is required for resistance against *T. gondii* in cultured murine astrocytes by activating vacuole vesiculation [24]. A role for Irga6 in the induction of structures carrying LC3 close to disrupting *T. gondii* vacuoles upon IFNγ-treatment has also been suggested [24]. Similarly, Irgm3 induces *T. gondii* vacuole vesiculation and fusion with autophagosomes in macrophages activated *in vivo* [23]. Recently, siRNA targeting of Irga6 was shown to partially revert the IFNγ-induced growth inhibition of *C. trachomatis*, whereas siRNA-mediated knockdown of Irgb6, Irgd, Irgm1, Irgm2 or Irgm3 did not [28]. In support of these data, we uncover Irga6 as a major effector protein in IFNγ-induced elimination of *C. trachomatis*. Despite an accumulation of other IRG members at the *C. trachomatis* inclusion in Irga6−/− cells, *C. trachomatis* grow more efficiently than in WT MEFs. Similarly, an analysis of isolated Irga6−/− astrocytes showed a significant loss of resistance to *T. gondii*, even though Irgb6, Irgd, Irgm2 and Irgm3 still localized to the parasitophorous vacuole [24]. Additionally, *C. trachomatis* inclusions in Irga6−/− cells do not colocalize with LAMP1 and LC3, confirming an involvement of autophagy in restricting pathogen growth. In *T. gondii*, it has been proposed that autophagic sequestration follows damage to the vacuolar membrane containing the organism [23], but it is not yet known whether such damage or a molecular modification of the target membrane leads to autophagic uptake. The present study clearly shows that molecular remodelling of the chlamydial inclusion membrane in response to IFNγ stimulation promotes fusion with autophagosomes.

There is some evidence that GMS subfamily proteins (Irgm1, Irgm2 and Irgm3) are required for the normal function of Irga6 [16]. When overexpressed in cells not stimulated with IFNγ, Irga6 and Irgb6 intracellularly mislocalized and aggregated. This mislocalization could be corrected by co-expression of Irgm1, Irgm2 and Irgm3. Another line of evidence shows that IRG proteins accumulate on the *T. gondii* vacuole in a cooperative manner to regulate anti-parasitic Irga6 function [36]. The assembly of Irga6, Irgb6, Irgd, Irgm2 and Irgm3 on *C. trachomatis* inclusions in WT MEFs and the lack of Irgm1 colocalization observed in this study is largely consistent with IRGs after infection of murine astrocytes with *T. gondii* [24]. The function of Irgm1, however, does not necessarily require its direct association with the microbial vacuole. Indeed, Irgm1-deficient mice are susceptible to *C. trachomatis* [37], *T. gondii* [38], *T. cruzi* [39] and *M. tuberculosis* [19] infection. The role of the other IRGs may be to induce the accumulation of effective Irga6 concentrations and/or its correct positioning for autophagosome remodelling. Recent work has demonstrated an essential role for Atg5, independent of autophagy, in trafficking Irga6 to vacuole membranes of *T. gondii* and subsequent pathogen clearance [40]. Here, we also found recruitment of Irga6 was linked to Atg5, in an autophagy dependent manner, as accumulation of Irga6 at early chlamydial inclusions was blocked in Atg5−/− MEFs. Future experiments will define the importance of these factors in recruitment of Irga6 to the inclusion and its antimicrobial function.

The inhibitory effects of IFNγ on chlamydial replication have been extensively studied, revealing marked inconsistencies in chlamydial strain susceptibility and antichlamydial effector mechanisms, as demonstrated by discrepancies in the role of IRGs in controlling chlamydial infections. Both Irgm3 and Irgb10 were found to mediate resistance to *C. trachomatis* in cultures and in systemically infected mice [20]. Irgm2 and Irgb10 have been implicated in *C. psittaci* resistance in cell cultures and locally infected mice [41]. In agreement with our study, Nelson and co-workers suggested a crucial role for Irga6 in *C. trachomatis* growth control [28]. In contrast, it was recently shown that Irga6−/− MEFs are not defective, but more efficient in restricting growth of *C. trachomatis* compared with control IFNγ-treated MEFs [37]. Subcellular localization studies only partly agree with our data, showing Irga6 localization and the absence of Irgm1 localization to inclusions upon IFNγ stimulation [20]. Also, Bernstein-Hanley and coworkers detected no Irgm3 at the *C. trachomatis* inclusion and the bacterium's growth in systemically infected mice was not affected; it remains unclear why resistance is not affected in Irga6−/− mice. More specific infection of the uterine mucosa by intrauterine inoculation with human chlamydial strains, as previously described [42], might lead to a more coherent outcome. Overall, these studies point to the complexity and diversity of IRGs that participate in host resistance mechanisms. The pleiotropic signaling capabilities and host and tissue specificities of IFNγ [43], the genomic differences among chlamydial strains studied [44], [45], differences in susceptibility among inbred mouse strains and the inherent experimental variation between laboratories, may account for these discrepancies.

It is indisputable that *C. muridarum* possesses a very effective mechanism to evade the murine IFNγ response, unlike *C. trachomatis*; however, the underlying mechanism remains largely hypothetical. Nelson and co-workers suggested a gene in the plasticity zone of *C. muridarum*, which is absent in *C. trachomatis*, is responsible for avoiding the Irga6-mediated growth inhibition by *C. muridarum* in murine cells [28]. This gene encodes a relatively large protein with a homology to a clostridial toxin and the *Yersinia* YopT virulence factor. YopT acts as cysteine protease that can inactivate Rho GTPase by the cleavage of the GTPase and its subsequent release from the membrane. Although indirect, the authors suggested that a *C. muridarum* hypothetical large toxin inactivates Irga6 by a similar mechanism. In contrast to our work, a recent study demonstrated the transient overexpression of Irgb10 in the absence of IFNγ was sufficient to reduce the yield of *C. trachomatis*, but not *C. muridarum* [37]. Overexpressed Irgb10 was found associated with *C. trachomatis* inclusions only. Based on this differential subcellular localization of Irgb10 in infected cells, the authors proposed Irgb10 is recruited to the inclusion to induce bacterial growth blockage. They also suggested that *C. muridarum* is protected from IFNγ-induced immune response by a mechanism that restricts access of Irgb10 to its inclusion. Here we show IFNγ-stimulated association of different IRG proteins (Irga6, Irgb6, Irgd, Irgm2 and Irgm3) with inclusions harbouring *C. trachomatis*, but not *C. muridarum*. Importantly, Irga6 was found to be the critical effector protein responsible for immune resistance to *C. trachomatis*, while other IRGs could have cooperative interactions. Cells deficient in Irga6 were highly permissive to *C. trachomatis* infection, although other IRGs were recruited in response to IFNγ. However, *C. muridarum* inclusions did not associate with any of these IRGs. These results strongly indicate that *C. muridarum* can prevent, by a yet undefined mechanism, not only the access and/or the activity of the effector Irga6, but also the localization of the so called ‘co-operative’ IRGs required for the anti-bacterial function of Irga6.

Our data indicate that modification of the inclusion is critical to the outcome of the host-parasite interaction; the presence of Irga6 on the inclusion membrane defeats the complex array of processes by which *C. trachomatis* seeks to delay phagosomal maturation. Further work will now unravel the precise mechanism(s) through which Irga6 promotes IFNγ -induced *C. trachomatis* elimination and *C. muridarum* uses to escape the murine IFNγ-induced response.

## Methods

### Chemicals and antibodies

RPMI-1640 medium and Dulbecco's minimal essential medium (DMEM) were purchased from Gibco-Invitrogen (Karlsruhe, Germany). Cycloheximide was obtained from Calbiochem (Darmstadt, Germany). IFNγ was purchased from Strathmann Biotec Gmbh. (Hamburg, Germany). Bafilomycin A1 (Baf A1) was purchased from Calbiochem. Rapamycin (Rapa) was obtained from Sirolimus LC Laboratories (Massachusetts, USA). Goat antibodies raised against mouse Irgb6 (clone A-20), Irgm2 (clone L-15), Irgm3 (clone Y-16), and Irgm1 (clone A-19), in addition to the rabbit antibody against mouse Irgd (clone M-85), were purchased from Santa Cruz Biotechnology, Inc. (Heidelberg, Germany). Other serological reagents used were: monoclonal anti-mouse Irga6 antibody raised in mice (clone 10D7 [46]), rat anti-mouse lysosomal associated membrane protein 1 (LAMP1) (clone 1D4B; Abcam, Cambridge, UK), monoclonal antibody to light chain 3 (LC3) (clone 5F10; purchased from nanoTools, Teningen, Germany), mouse monoclonal anti-human β-actin (Sigma-Aldrich), rabbit polyclonal anti-*Chlamydia* genus-specific antibody (Milan Analytica AG, La Roche, Switzerland), and mouse monoclonal anti-*C. trachomatis* hsp60 (Alexis, Loerrach, Germany) and LPS (clone CF6J12, Abcam, Cambridge, UK). The IMAGEN kit for detection of *Chlamydia* was from DAKO (Hamburg, Germany). Secondary labeled antibodies for immunofluorescence and Western analyses were purchased from Molecular Probes- MoBiTech (Goettingen, Germany), Dianova (Hamburg, Germany), BD Biosciences-Pharmingen (Heidelberg, Germany), or Amersham (NJ, USA).

### *Chlamydia trachomatis* and *C. muridarum* propagation and murine cell cultures

*C. trachomatis* Lymphogranuloma venereum (LGV) serovar L2 and *C. muridarum*, a generous gift from Konrad Sachse (Friedrich-Loeffler-Institut, Federal Research Institute for Animal Health, Jena, Germany) were routinely propagated in HeLa cells grown in RPMI-1640 medium supplemented with glutamine and 5% fetal bovine serum (FBS). *Chlamydia* culturing, preparation of EB stock, and estimation of inclusion forming units (IFU)/ml were conducted as previously described [47]. Wild type (WT) MEFs and autophagy-deficient (Atg5-knockout) MEFs were generously provided by Noburo Mizushima (Department of Physiology and Cell Biology, Tokyo Medical and Dental University, Tokyo Japan). Irga6-lacking MEFs were generated in-house (see below). MEFs were grown in DMEM provided with 10% FBS, 200 mM glutamine and 1 mM sodium pyruvate, and incubated at 37°C and 5% CO_2_ in a humidified tissue culture chamber.

### Infection of MEFs

MEFs were seeded onto 6-well-plates and incubated overnight to allow adherence. Host cells were then infected with either *C. trachomatis* or *C. muridarum* diluted in cell growth medium at an MOI of 1 and incubated at 35°C and 5% CO_2_. Two hours post infection (p.i.) cells were washed and loaded with fresh medium containing 100 or 200 U/ml IFNγ. Control infected cells were not treated with the cytokine. Cell cultures were then incubated as above for 48 h.

### Infectivity titration assays

Formation of infectious *C. trachomatis* and *C. muridarum* progeny in a secondary infection was assessed as described elsewhere [47]. Briefly, infected cells were mechanically destructed using glass beads. Lysates were serially diluted in IM and inoculated onto HeLa cells for 2 h. Cells were then washed and further incubated for 24 h in RPMI medium with 5% FBS and 1 µg/ml cycloheximide. Chlamydial inclusions were fixed with methanol and then immunostained with IMAGEN *Chlamydia* kit. Subsequently, inclusions were visualized and counted using immunofluorescence microscopy (Nikon), and infectivity of progeny was expressed as IFU/ml.

### Generation of Irga6-deficient MEFs

MEFs were prepared as described previously [48]. Briefly, embryos generated from Irga6−/−×C57BL/6 and Irga6−/−×Irga6−/− crosses were isolated on day 13.5 of development. Placenta, membranes, visceral tissues and the head were removed from embryos and the remaining tissue was minced and trypsinized to produce single cells. Single cells were passaged twice in DMEM containing 10% FBS and then stored in liquid nitrogen for later use.

### Transfection of host cells

MEFs were seeded onto coverslips in 12-well-plates and incubated overnight at 37°C and 5% CO_2_ to allow adherence. MEFs were then washed once and transfected with 1 µg/ml plasmid DNA encoding pEGFP-rat LC3 (a kind gift from Tamotsu Yoshimori, Department of Cellular Regulation, Research Institute for Microbial Diseases, Osaka University, Osaka, Japan), using Lipofectamine 2000 (Gibco-Invitrogen) according to manufacturer's instructions. One day post-transfection, cells were stimulated for 24 h with 100 U/ml IFNγ or left unstimulated (control cells) and incubated in a humidified cell culture incubator. Next, transfected cells were infected with *C. trachomatis* or *C. muridarum* at a MOI of 5 and incubated for time periods as indicated at 35°C and 5% CO_2_, before processing for confocal microscopy and Western blotting. For colocalization studies MEFs were stained 3 h p.i. (MOI 5), facilitating the detection of a large number of intracellular bacteria before eradication in response to IFNγ.

### Fluorescence confocal microscopy

MEFs were plated onto coverslips in 12-well-plates at a density of 2×10^5^ cells per well and incubated overnight. Next, cells were treated with 100 U/ml IFNγ for 24 h before infection or left untreated as a control. MEFs were then inoculated with *C. trachomatis* at a MOI of 5 and incubated at 35°C and 5% CO_2_. Two hours p.i., cells were washed, provided with fresh medium with or without IFNγ, and incubated as before. At indicated time points, cells were fixed and permeabilized with ice-cold methanol for 5 min at room temperature (RT). In some experiments, cells were fixed for 25 min with 4% paraformaldehyde at RT, permeabilized with either 0.2% saponin, 0.3% Triton-100× or digitonin and blocked using 2.5% BSA. Cells were sequentially incubated for 1 h at RT with primary and secondary antibodies. Then, coverslips were mounted onto glass slides using Mowiol and examined by Leica TCS-SP laser scanning confocal microscope equipped with a krypton/argon laser. Photomicrographs were processed using Adobe Photoshop 6.0 (Adobe Systems) and Microsoft Power-Point.

### Immunoblotting

MEFs were stimulated with IFNγ, as indicated above, or with the classical autophagy inducer Rapa at a concentration of 100 nM for 2 h. In some experiments, unstimulated and IFNγ-stimulated cells were infected with *C. trachomatis* or *C. muridarum* for either 3 or 8 h. Cells were washed with cold PBS, and lysed for 30 min on ice in Triton-100× lysis buffer (20 mM Tris-HCl; pH 7.6, 150 mM NaCl, 1% Triton-100X), containing 2 mM PMSF and complete protease inhibitor cocktail (Roche). The lysates were then centrifuged at 8,000×g for 10 min. Equal amounts of proteins were subjected to 12% sodium dodecyl sulfate-polyacrylamide gel electrophoresis (PAGE). Protein bands were transferred electrophoretically onto Immobilon-P polyvinylidene difluoride membranes (Millipore, MA, USA). The membranes were blocked with 5% fat-skim milk in TBS (Tris-buffered saline; pH 7.5), containing 0.05% Tween-20 for 1 h at RT. Next, membranes were incubated with the monoclonal mouse anti-LC3 (overnight, at 4°C) or anti-β-actin (1 h at RT) diluted in TBS-0.05% Tween-20. Membranes were washed and then incubated with secondary antibodies conjugated with horseradish peroxidase. Signal detection was performed with the enhanced chemiluminescence system (ECL, Amersham).

### Inhibition of lysosomal acidification

MEFs were seeded onto coverslips and exposed to IFNγ for 23 h. MEFs were pretreated with 100 nM Baf A1 for 1 h before the infection. Cells were then inoculated with *C. trachomatis* (MOI of 5), and continuously incubated with Baf A1 throughout the experiment period. Control cell monolayers were treated with dimethylsulfoxide, in which Baf A1 was dissolved. Specimens were then fixed and double stained for LAMP1 and *C. trachomatis*. The percentages of colocalization of chlamydial inclusions with LAMP1 were determined in the presence or absence of the inhibitor.

To examine the effect of the acidification inhibition on the recovery of infectious chlamydial progeny, cells were grown in 6-well-plates, treated, and infected as mentioned above. Cells were then harvested 2 days p.i., lysed and diluted, and formation of infectious *C. trachomatis* was assessed by infectivity titration assays on fresh HeLa cell cultures.

### Assessment of host cell viability

Lactate dehydrogenase (LDH) colorimetric assay (Roche Diagnostics, Mannheim, Germany) was used according to manufacturer's instructions. This assay is based on the measurement of LDH activity released from the cytosol of damaged cells into the supernatant.

## Supporting Information

Figure S1 No colocalization of Irga6, Irgb6, Irgd, Irgm1, Irgm2 and Irgm3 at early *C. muridarum* inclusions upon IFNγ stimulation. Double immunofluorescence labelling of IRGs and *C. muridarum* in WT (A), Atg5 −/− (B) and Irga6−/− (C) MEFs stimulated for 24 h with 100 U/ml IFNγ and then infected for 3 h with *C. muridarum* (MOI 5). IFNγ untreated MEFs were similarly infected. Upon IFNγ induction all tested IRGs except Irgb6 were highly expressed in all cell lines (no Irga6 in Irga6 −/− MEFs) without any colocalization to bacterial inclusions. Scale bar represents 5 µm
(2.84 MB TIF)

Figure S2 IFNγ cannot induce autolysosomal fusion with early *C. muridarum* inclusions to suppress bacterial growth in WT, Atg5−/− and Irg6−/− MEFs. (A) *C. muridarum* inclusions avoid interaction with lysosomes in all MEFs tested despite IFNγ induction. IFNγ treated and untreated Irga6, Atg5−/− and WT MEFs were infected with *C. muridarum* as in Fig. 3A and B. (B) Quantification of LAMP1-positive chlamydial inclusions revealed insignificant colocalization rates. Percentage of colocalization depicted. Error bars ±SD, n = 3. (C) No maturation of bacterial inclusions into autophagosomes. WT, Irga6−/− and Atg5−/− MEFs were first transfected for 24 h with the autophagosome membrane marker GFP-LC3 and then exposed to 100 U/ml IFNγ for an additional 24 h. Next, cells were infected with *C. muridarum* (MOI 5) for 8 h. IFNγ-untreated control cells were similarly infected 48 h post-transfection. LC3 (green) bacterial inclusions (red) with very low processing for the LC3 (D) Quantification of GFP-LC3-positive *C. muridarum* inclusions demonstrate insignificant colocalization rates among different treatments. For quantification of both LAMP1 and GFP-LC3 +ve inclusions, around 300 inclusions were examined. Colocalization expressed as a mean percentage: number of GFP-LC3 +ve or LAMP1 +ve inclusions, respectively / total number of inclusions ×100. Error bars ±SD, n = 3. Scale bar represents 5 µm
(1.37 MB TIF)

Figure S3 Irgd, Irgm2, Irgm3, Irgb6 and Irgm1 show a similar interaction pattern with *C. trachomatis* early inclusions in Irga6-knockout MEFs under stimulation with IFNγ. Irga6-deficient cells were exposed to the cytokine, infected with the pathogen, and stained for Chlamydia and IRGs exactly as described in the legend to Fig. 2. Untreated knockout cells were infected and stained in parallel for comparison reasons. (A) Confocal micrographs showing the double labelling of cells with antibodies against the pathogen and different IRGs. (B) Quantification of IRG colocalization with *C. trachomatis* inclusions. Around 300 bacterial inclusions examined. Colocalization expressed as a mean percentage: number of IRG +ve inclusions / total number of inclusions ×100. Error bars ±SD, n = 3. Scale bar represents 5 µm.
(0.67 MB TIF)

Figure S4 Irga6 does not play a role in the regulation of the host autophagic machinery during *C. muridarum* infection. (A–C) Anti-LC3 immunoblot analysis of total lysates from uninfected WT, Atg5−/− and Irga6−/− MEFs or from cultures infected for indicated time points. Some uninfected cell cultures were exposed to 100 nM Rapa for 3 h or to 100 U/ml IFNγ for 32 h. Other monolayers were pretreated with IFNγ for 24 h prior to infection and then infected in the presence of chemicals. 1–7 indicate the different treatments. Autophagy induction is reflected by the increased cellular level of LC3 and formation of autophagosome-associated LC3-II. Host β-actin was used to control equal loading of proteins. After infection with *C. muridarum*, LC3 II level increases but to a less extent than *C. trachomatis* (Fig. 7) in IFNγ treated (A) WT cells. LC3 levels do not increase in IFNγ treated (C) Irga6−/− cells. (B) Defective autophagy in Atg5−/− MEFs was observed, indicated by absence of LC3 processing.
(0.39 MB TIF)
