# Supplementary figures and images for: IFN-γ-Inducible Irga6 Mediates Host Resistance against Chlamydia trachomatis via Autophagy
Source: PLoS One. 2009 Feb 26;4(2):e4588. doi: 10.1371/journal.pone.0004588 (PMC2643846; doi:10.1371/journal.pone.0004588)

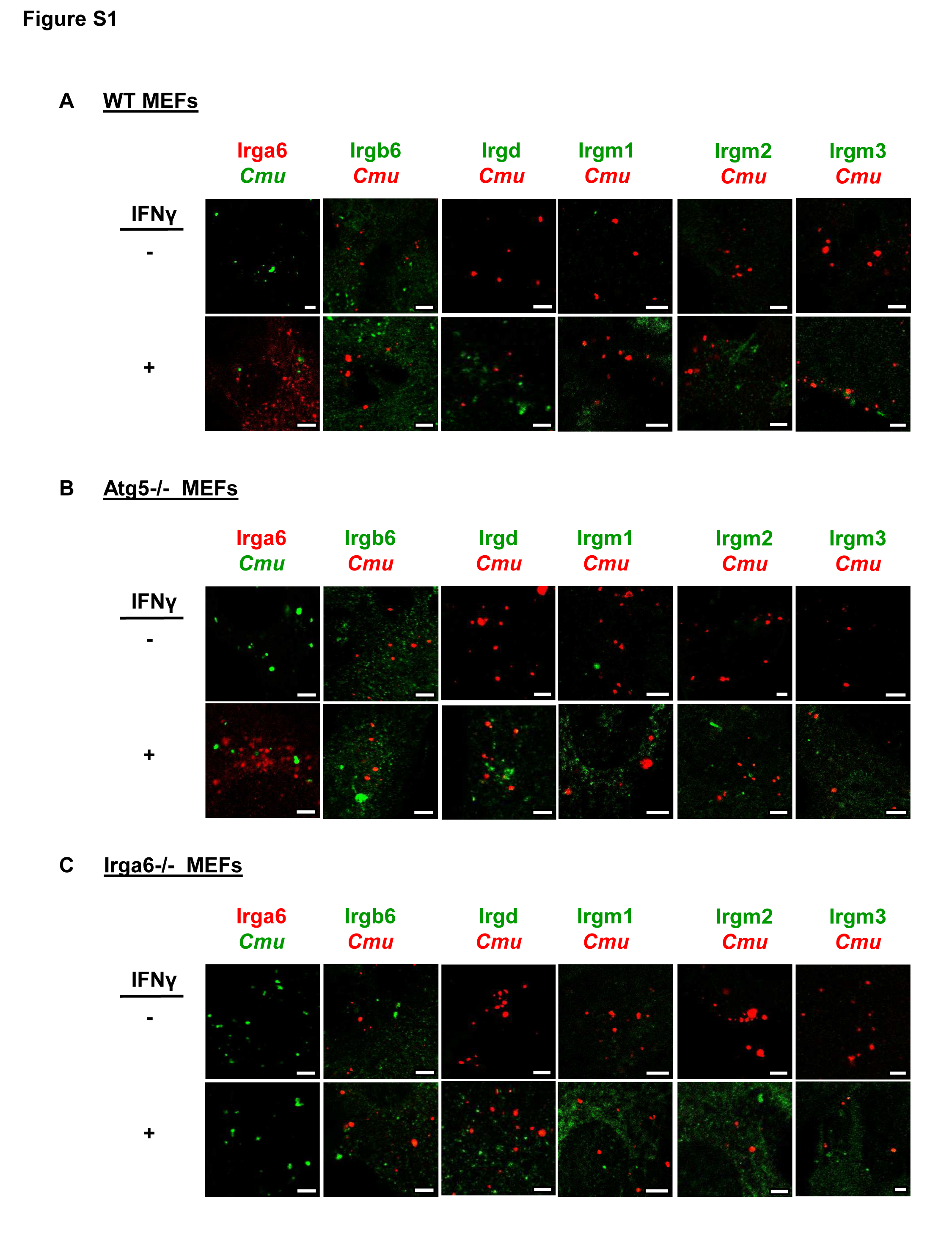

Supplement: Figure S1 — No colocalization of Irga6, Irgb6, Irgd, Irgm1, Irgm2 and Irgm3 at early C. muridarum inclusions upon IFNγ stimulation. Double immunofluorescence labelling of IRGs and C. muridarum in WT (A), Atg5 −/− (B) and Irga6−/− (C) MEFs stimulated for 24 h with 100 U/ml IFNγ and then infected for 3 h with C. muridarum (MOI 5). IFNγ untreated MEFs were similarly infected. Upon IFNγ induction all tested IRGs except Irgb6 were highly expressed in all cell lines (no Irga6 in Irga6 −/− MEFs) without any colocalization to bacterial inclusions. Scale bar represents 5 µm (2.84 MB TIF) [file pone.0004588.s001.tif]

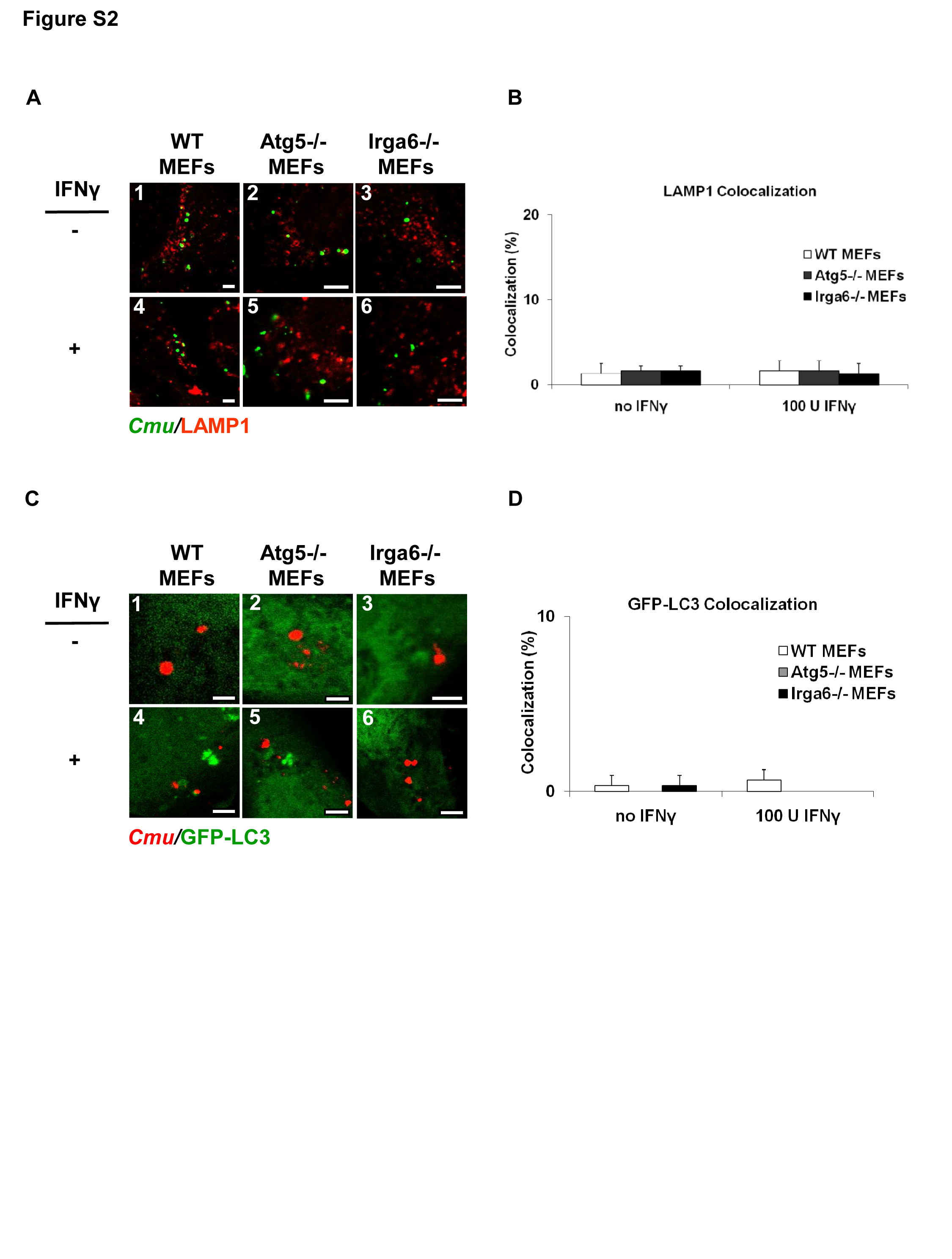

Supplement: Figure S2 — IFNγ cannot induce autolysosomal fusion with early C. muridarum inclusions to suppress bacterial growth in WT, Atg5−/− and Irg6−/− MEFs. (A) C. muridarum inclusions avoid interaction with lysosomes in all MEFs tested despite IFNγ induction. IFNγ treated and untreated Irga6, Atg5−/− and WT MEFs were infected with C. muridarum as in Fig. 3A and B. (B) Quantification of LAMP1-positive chlamydial inclusions revealed insignificant colocalization rates. Percentage of colocalization depicted. Error bars ±SD, n = 3. (C) No maturation of bacterial inclusions into autophagosomes. WT, Irga6−/− and Atg5−/− MEFs were first transfected for 24 h with the autophagosome membrane marker GFP-LC3 and then exposed to 100 U/ml IFNγ for an additional 24 h. Next, cells were infected with C. muridarum (MOI 5) for 8 h. IFNγ-untreated control cells were similarly infected 48 h post-transfection. LC3 (green) bacterial inclusions (red) with very low processing for the LC3 (D) Quantification of GFP-LC3-positive C. muridarum inclusions demonstrate insignificant colocalization rates among different treatments. For quantification of both LAMP1 and GFP-LC3 +ve inclusions, around 300 inclusions were examined. Colocalization expressed as a mean percentage: number of GFP-LC3 +ve or LAMP1 +ve inclusions, respectively / total number of inclusions ×100. Error bars ±SD, n = 3. Scale bar represents 5 µm (1.37 MB TIF) [file pone.0004588.s002.tif]

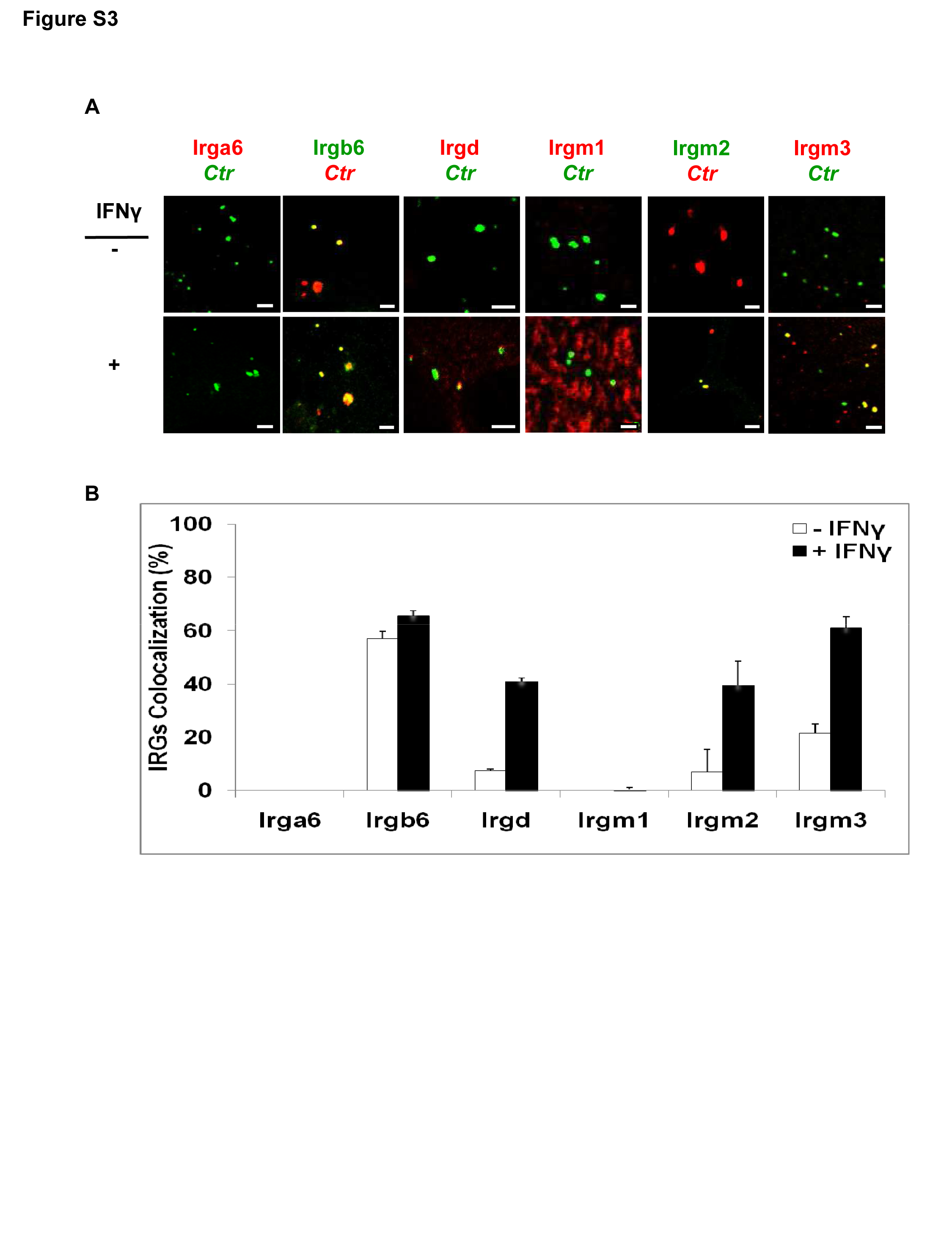

Supplement: Figure S3 — Irgd, Irgm2, Irgm3, Irgb6 and Irgm1 show a similar interaction pattern with C. trachomatis early inclusions in Irga6-knockout MEFs under stimulation with IFNγ. Irga6-deficient cells were exposed to the cytokine, infected with the pathogen, and stained for Chlamydia and IRGs exactly as described in the legend to Fig. 2. Untreated knockout cells were infected and stained in parallel for comparison reasons. (A) Confocal micrographs showing the double labelling of cells with antibodies against the pathogen and different IRGs. (B) Quantification of IRG colocalization with C. trachomatis inclusions. Around 300 bacterial inclusions examined. Colocalization expressed as a mean percentage: number of IRG +ve inclusions / total number of inclusions ×100. Error bars ±SD, n = 3. Scale bar represents 5 µm. (0.67 MB TIF) [file pone.0004588.s003.tif]

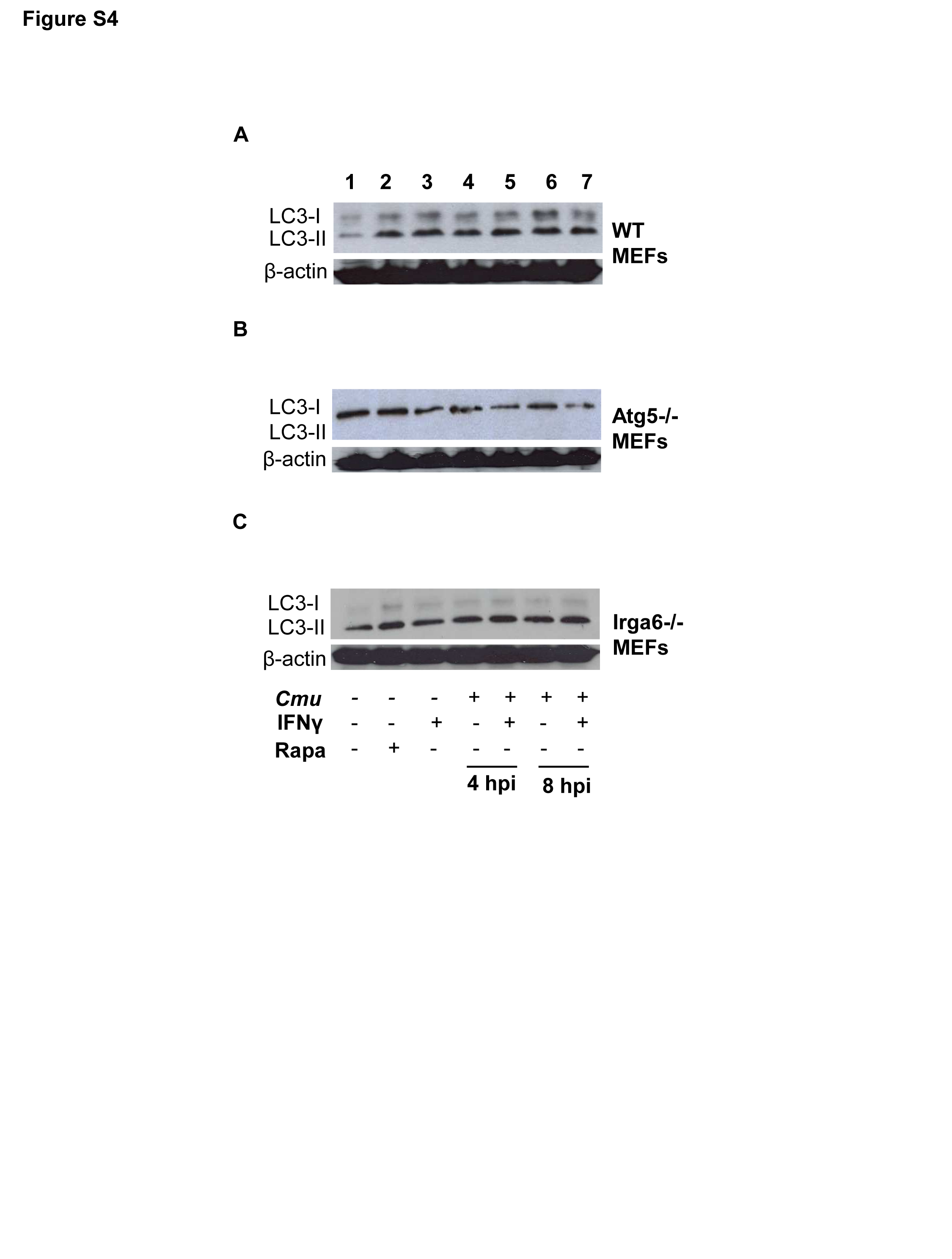

Supplement: Figure S4 — Irga6 does not play a role in the regulation of the host autophagic machinery during C. muridarum infection. (A–C) Anti-LC3 immunoblot analysis of total lysates from uninfected WT, Atg5−/− and Irga6−/− MEFs or from cultures infected for indicated time points. Some uninfected cell cultures were exposed to 100 nM Rapa for 3 h or to 100 U/ml IFNγ for 32 h. Other monolayers were pretreated with IFNγ for 24 h prior to infection and then infected in the presence of chemicals. 1–7 indicate the different treatments. Autophagy induction is reflected by the increased cellular level of LC3 and formation of autophagosome-associated LC3-II. Host β-actin was used to control equal loading of proteins. After infection with C. muridarum, LC3 II level increases but to a less extent than C. trachomatis (Fig. 7) in IFNγ treated (A) WT cells. LC3 levels do not increase in IFNγ treated (C) Irga6−/− cells. (B) Defective autophagy in Atg5−/− MEFs was observed, indicated by absence of LC3 processing. (0.39 MB TIF) [file pone.0004588.s004.tif]
